# Posterior Cortical Atrophy: Characteristics From a Clinical Data Registry

**DOI:** 10.3389/fneur.2020.00358

**Published:** 2020-06-03

**Authors:** Jennifer J. Olds, William L. Hills, Judith Warner, Julie Falardeau, Lori Haase Alasantro, Mark L. Moster, Robert A. Egan, Wayne T. Cornblath, Andrew G. Lee, Benjamin M. Frishberg, Roger E. Turbin, David M. Katz, John A. Charley, Victoria S. Pelak

**Affiliations:** ^1^Department of Ophthalmology, Keesler Air Force Base, Biloxi, MS, United States; ^2^Department of Neurology & Ophthalmology, Casey Eye Institute, Oregon Health & Science University, Portland, OR, United States; ^3^Department of Ophthalmology & Neurology, John A. Moran Eye Center, University of Utah, Salt Lake City, UT, United States; ^4^Department of Ophthalmology, Casey Eye Institute, Oregon Health & Science University, Portland, OR, United States; ^5^Department of Neuroscience, The Neurology Center of Southern California, University of California San Diego School of Medicine, Carlsbad, CA, United States; ^6^Department of Neurology and Ophthalmology, Wills Eye Hospital and Thomas Jefferson University, Philadelphia, PA, United States; ^7^Eye & Vascular Neurology, LLC, Carlton, OR, United States; ^8^Department of Ophthalmology, Visual Sciences & Neurology, W.K. Kellogg Eye Center, University of Michigan, Ann Arbor, MI, United States; ^9^Department of Ophthalmology, Blanton Eye Institute, Houston Methodist Hospital, Houston, TX, United States; ^10^Division of Neuro-ophthalmology and Orbital Surgery, Rutgers New Jersey Medical School, Institute of Ophthalmology and Visual Sciences, Newark, NJ, United States; ^11^Bethesda Neurology, LLC, Department of Ophthalmology & Neurology, Howard University Hospital, Georgetown University Hospital, Washington, DC, United States; ^12^Retired Private Practice Ophthalmologist, Pittsburgh, PA, United States; ^13^Department of Neurology & Ophthalmology, UCHealth Sue Anschutz-Rodgers Eye Center and the Neurosciences Center, University of Colorado School of Medicine, Aurora, CO, United States

**Keywords:** posterior cortical atrophy, Benson's syndrome, higher-order visual dysfunction, Alzheimer's disease, neuro-ophthalmology, patient registry

## Abstract

**Background:** Posterior cortical atrophy (PCA) is a neurodegenerative syndrome that presents with higher-order visual dysfunction with relative sparing of memory and other cognitive domains, and it is most commonly associated with Alzheimer's disease pathology. There is a lack of data regarding the presentation of PCA to non-cognitive specialists. Therefore, we collected clinical data from neuro-ophthalmologists regarding the presentation of PCA to their practices and compared data to published cohorts and a published survey of cognitive specialists.

**Methods:** Members of the North American Neuro-Ophthalmology Society Listserv (NANOSnet) were invited to complete an online, retrospective, chart-review data-entry survey regarding their patients with PCA, and REDCap was used for data collection.

**Results:** Data for 38 patients were entered by 12 neuro-ophthalmologists. Patient mean age at presentation was 67.8 years, and 74% of patients were women. Difficulty reading was reported at presentation by 91% of patients, and poor performance on color vision, stereopsis, and visual field testing (performed reliably by 36/38 patients) were common findings. Most patients who were treated were treated with donepezil and/or memantine.

**Conclusions:** Compared to published data from cognitive specialists, patients presenting to neuro-ophthalmology with PCA were more likely to be older and female and have a reading complaint. Reliable visual field testing was the norm with homonymous defects in the majority of patients. The neuro-ophthalmologist plays an important role in diagnosing PCA in older adults with unexplained visual signs and symptoms, and future studies of PCA should involve multiple specialists in order to advance our understanding of PCA and develop effective treatments.

## Introduction

Posterior cortical atrophy (PCA) is a progressive, neurodegenerative syndrome that is characterized by higher-order visual dysfunction with initial relative sparing of memory and other cognitive functions (1). The prevalence of PCA is unknown, and it is commonly considered an atypical presentation of Alzheimer's disease (AD) because most cases at autopsy reveal AD pathology. Other pathologies found in isolation or with Alzheimer's pathology are α-synucleinopathy consistent with Lewy body dementia, corticobasal degeneration, prion disease, and non-specific pathologies (1). In 2017, formal PCA diagnostic criteria were agreed upon by expert consensus and published with the intention of creating a uniform definition to be used in research settings (2). See Table 1 for a summary of the criteria. Since the initial description by Benson et al. (3), PCA patient characteristics have been described in numerous small case series and case reports, and these reveal that ocular disease is not the cause of visual symptoms in PCA and that the early stages of the PCA syndrome are distinct from AD by the clinical complaints, the type of cognitive and perceptual dysfunction present, and the neuroimaging findings (2). Posterior cortical atrophy typically progresses to a multicognitive domain dementia, and the initial features that distinguish the syndrome from typical AD, or typical Lewy body dementia, blur later in the course (3). Other patients with PCA will have progressive higher-order visual dysfunction with persistent relative sparing of other cognitive domains over many years and even beyond a decade (4).

**Table 1:** Consensus research framework criteria for posterior cortical atrophy (2).

Core Clinical Features: All three must be present	•Insidious onset •Gradual progression •Prominent early disturbance of visual functions, other posterior cognitive functions, or both
Core Cognitive Features: At least three must be present as an early or presenting feature	Space perception deficit, simultanagnosia, object perception deficit, constructional dyspraxia, environmental agnosia, oculomotor apraxia, dressing apraxia, optic ataxia, alexia, left/right disorientation, acalculia, limb apraxia (not limb-kinetic), apperceptive prosopagnosia, agraphia, homonymous visual field defect, finger agnosia
Core Neuroimaging Features: Supportive of diagnosis	Predominant occipitoparietal or occipitotemporal atrophy or hypometabolism or hypoperfusion on MRI, FDG-PET, or SPECT
Non-posterior Cortical Features: All of the following must be evident	•Relatively spared anterograde memory function •Relatively spared speech and non-visual language functions •Relatively spared executive functions •Relatively spared behavior and personality
Exclusions:	•No afferent visual dysfunction or afferent lesions to explain symptoms •No vascular lesions to explain symptoms •No brain tumor or other mass lesion to explain symptoms •No evidence of other causes to explain symptoms

Investigations of disorders that are relatively rare and/or poorly recognized, such as PCA, face many challenges and require collaboration across institutions for true progress to be made in understanding clinical presentations, risk factors, and pathophysiology, as well as for the development of treatments (5). Many patients with PCA are diagnosed after significant delays following onset of symptoms, and it is suspected that the early age at onset and a prolonged search for ocular causes of visual symptoms lead to the delay in diagnosis (1, 6).

The recent consensus classification criteria for PCA for research purposes, set forth by an international, multidisciplinary working party as noted (2), have accelerated the pace of publications on PCA. Experts in the working party were members of the Atypical Alzheimer's Disease and Associated Disorders Professional Interest Area (now called the Atypical AD PIA) of the Alzheimer's Association International Society to Advance Alzheimer's Research and Treatment (ISTAART). Criteria were based on consensus following a survey-based questionnaire that queried experts regarding their experience in the diagnosis and care of patients with PCA, and the experts included behavioral neurologists, neuropsychologists, psychiatrists, and a neuro-ophthalmologist/behavioral neurologist (2).

A recent study of the longitudinal cognitive and imaging profiles from 117 patients with PCA, compared to typical AD, provided new insights into the PCA presentation and course within a tertiary dementia center in the United Kingdom and two other centers in Spain and the United States (7). A recent multicenter genetic study with just over 300 patients with PCA from 11 centers worldwide demonstrates how important collaborative efforts are in generating larger cohorts to better understand PCA and similar syndromes (8). Nonetheless, knowledge about the PCA syndrome continues to rely on patients from large tertiary care centers with extensive research programs that focus on PCA and/or atypical presentations of AD (i.e., non-amnestic clinical phenotypes of AD, for instance) or small case series of patients. Minimal data exist regarding the presentation of PCA to the neuro-ophthalmologist, other than single case reports or small series of PCA patients (previously referred to as the visual variant of AD) presenting to the neuro-ophthalmologist with homonymous visual field abnormalities on threshold perimetry (9, 10).

The utility of studying atypical presentations of AD should not be underestimated given that atypical AD represents ~25% of the more than 5 million people living with AD in the United States (11). Arguably, the study of focal cortical presentations could contribute to our understanding of disease “spread” in the central nervous system and help clarify relationships between clinical manifestations and the interaction of amyloid and tau pathology.

Given this background, we aimed to describe the clinical presentation of PCA to neuro-ophthalmologists using patient data collected across multiple institutions and community practices by a secure, online data registry, and compare findings to published studies of PCA and to survey data from the ISTAART Atypical AD PIA cognitive experts.

## Methods

After institutional review board approval, an introductory letter, and a link to an online, chart review, clinical data survey was emailed to volunteers from NANOSnet (North American Neuro-Ophthalmology Society email Listserv) members. The survey was designed following a literature review to identify the salient signs and symptoms previously reported for the presentation of PCA, and participating neuro-ophthalmologists were instructed to gather patient data via retrospective chart reviews. The patient data were captured using the REDCap platform (*R*esearch *E*lectronic *D*ata *Cap*ture), which is a secure web application that is compliant with Health Insurance Portability and Accountability Act, 21CFR part 11, FISMA (low, moderate, high), and international ethical standards. Posterior cortical atrophy was defined using the criteria of Tang-Wai et al. (12), because the survey was developed prior to the publication of the 2017 research criteria. Variables collected included certain patient identifiers to prevent duplicate entries, demographics, presenting and past medical histories, evaluations, and treatments. A list of 18 symptoms (Table 2) was presented to the physician respondent to indicate the presence of symptoms at *presentation, later in the disease course, never developed*, or *unsure*. For some symptoms, lay terminology was used. For others, medical terms were used for the sake of brevity, yet still interpreted as patient-reported symptoms, for example, simultanagnosia or apperceptive agnosia. Similarly, for signs on initial examination, a list was presented for physician respondents to indicate the presence at *presentation, later in the disease course, never developed*, or *unsure*. Branching logic helped determine additional details, for example, which color plate test was used. Data regarding diagnostic testing utilized, treatment, and subjective assessment of treatment outcomes were also collected. All sections included an “other” choice with the opportunity to provide written responses using free text. All fields were optional to account for missing clinical data. Survey responses were tabulated. Bivariate correlation analysis was performed to evaluate associations with age.

**Table 2:** Survey questions regarding initial symptoms at presentation (for survey questions regarding signs, see manuscript and Figure 1).

With what symptoms did your patient initially present?
1. Blurry vision
2. Difficulty reading
3. Difficulty with depth perception
4. Environmental disorientation
5. Visual hallucinations
6. Alexia
7. Apperceptive visual agnosia
8. Dressing apraxia
9. Limb apraxia
10. Memory difficulty
11. Prosopagnosia
12. Acalculia
13. Agraphia
14. Left/right disorientation
15. Finger agnosia
16. Anomia
17. Parkinsonism
18. Constructional dyspraxia
19. Other (please specify or describe above)

## Results

A total of 12 neuro-ophthalmologists from 11 institutions and community practices within the United States completed surveys for 38 patients. The last survey data were collected in 2017, and more than 97% of survey data were completed within the first 12 months between 2015 and 2016. Physicians were board-certified ophthalmologists (five) or neurologists (six) with subspecialty training in neuro-ophthalmology. The patient cohort was 74% female with a mean age of 67.8 ± 9.70 years and a median age of 69 years with a range of 45–81 years. Mean and median age for men was 69.6 ± 9.2 years and 71 years, respectively (range, 48–80 years), and mean and median age for women was 67.1 ± 7.5 years and 64 years, respectively (range, 52–81 years). Twenty-one percent of patients reported a history of traumatic brain injury (TBI), and 19% reported a family history of dementia of the AD type.

### Symptoms

**Figure 1:**
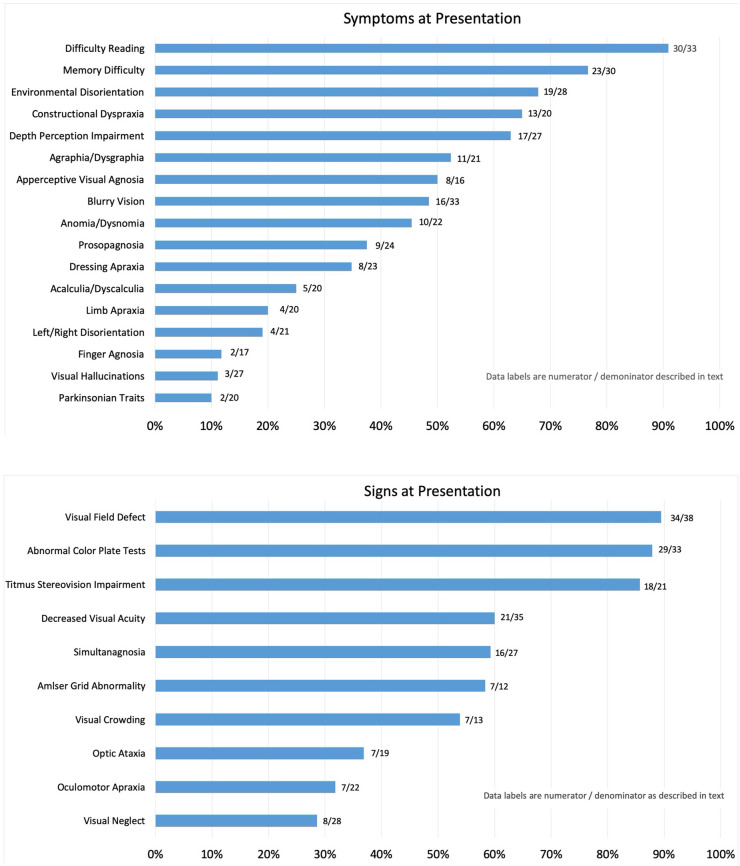
Reported symptoms **(Top)** and signs **(Bottom)** on presentation. Symptoms and signs are reported as a percentage of patients with PCA determined to have the symptom at initial presentation to the neuro-ophthalmologist.

The frequency of symptoms at presentation is noted as percentages in Figure 1. The numerator consists of items marked as “yes, on presentation,” and the denominator includes those marked as “no, but developed later in the course” plus “no, never developed” plus “no, not sure if developed later.” Responses that were “not sure, but did have later in the course” and “not sure” were not included in the denominator. This method was applied in order to report the symptoms that were definitively determined to be present or not at initial presentation and for most symptoms; none to four patients were excluded from the denominator. Exceptions included apperceptive visual agnosia (11 uncertain), finger agnosia (6 uncertain), and constructional dyspraxia (5 uncertain). The most frequent symptom reported by patients at presentation was “difficulty reading,” which included 91% of patient cases. Memory difficulty was reported by 77% of patients, and environmental disorientation was noted by 68%, whereas 65% reported symptoms consistent with constructional dyspraxia, 63% with difficulty with depth perception, 52% with agraphia/dysgraphia, and 50% with apperceptive visual agnosia. Symptoms reported at presentation by fewer than 50% of patients included blurry vision (48%), anomia (45%), prosopagnosia (38%), dressing apraxia (35%), acalculia (25%), limb apraxia (20%), left/right disorientation (19%), finger agnosia (12%), and visual hallucinations (11%). Parkinsonism was listed on the survey as a “symptom” with the intent to allow the respondents to interpret symptomatic complaints stemming from resting tremor, bradykinesia, rigidity, and postural instability as parkinsonian symptoms, and 10% of patients had symptoms of parkinsonian traits at presentation. Other symptoms written in by respondents included photophobia (four patients). Alexia, or marked loss or loss of ability to read, was a presenting symptom in 74% of patients; a second survey question asked whether patients complained simply of “reading difficulty,” and 91% of patients reported such.

### Signs

**Figure 2:**
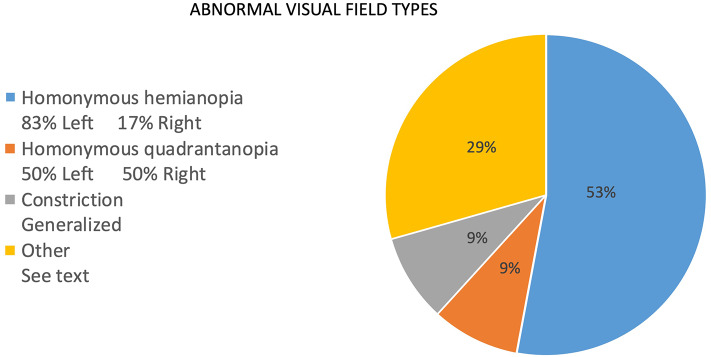
Types of visual field defects in patients with PCA at presentation to the neuro-ophthalmologist.

The frequency of signs reported at presentation is noted as percentages in Figure 2. For the same reasons as noted for report of symptoms, the numerator consists of items marked as “yes, on presentation,” and the denominator includes those marked as “no, but developed later in the course” plus “no, never developed” plus “no, not sure if developed later.” Responses that included “not sure, but did have later in the course” and “not sure” were not included in the denominator. Of the initial findings on examination, threshold perimetric visual field defects were present in 89% of the patients, with 62% revealing homonymous defects (Figure 2). The majority of homonymous defects were left-sided. Generalized constriction, which is a pattern revealing decreased peripheral vision with sparing of the central portions of the visual field, was present in 9% of the patients. Three patients had normal studies, and only two patients were unable to perform visual field testing reliably, as determined by the neuro-ophthalmologist. Color plate testing was abnormal in 88% of patients, and just over 50% of patients were tested with the Hardy–Rand–Rittler (HRR) pseudoisochromatic test plates and the remainder with Ishihara color plates. Just over half of the patients (52%) were unable to see the control color plate, and this was more commonly reported for those testing with HRR plates. Decreased Titmus stereopsis at near was noted in 86% of patients. Decreased visual acuity (i.e., best corrected visual acuity <20/20 either eye) was present in 60% of patients, simultanagnosia in 59%, and abnormalities noted on Amsler grid testing found in 58%. Central vision crowding (defined as better visual acuity performance with use of single letter compared to flanked letter testing) was noted in 54%. Optic ataxia was noted in 37%, oculomotor apraxia in 32%, and visual neglect in 29% of patients on initial examination. Uncertainty regarding the presence of signs at presentation was greater than that noted for symptoms, and this included 20 of 38 patients for crowding, 12 of 38 for optic ataxia, 12 of 38 for Titmus stereopsis, 11 of 38 for oculomotor apraxia, 5 of 38 for visual neglect, 5 of 38 for color plate testing, and 6 of 38 for simultanagnosia. The authors note that the uncertainty of these signs at presentation was due to lack of specific testing or documentation of testing at initial presentation.

### Evaluation

Thirty-five of 38 patients were reported as having magnetic resonance (MR) imaging of the brain, and two had computed tomography (CT) brain imaging. Of the reported findings on neuroimaging, generalized atrophy was most commonly found, and only one imaging report specifically noted the predominance of PCA. However, review of the brain imaging revealed that 34 of 38 CT brain scans by MR and CT had posterior atrophy (occipitoparietal, parietal–occipital–temporal, or both with or without mesiotemporal atrophy). Five were normal. A total of 12 patients underwent fluorodeoxyglucose (FDG) positron emission tomography (PET) imaging, and occipitoparietal hypometabolism was described in 50% (6/12), occipitoparietotemporal hypometabolism in 33% (4/12), and mesiotemporal hypometabolism in 17% (2/12). Cerebrospinal fluid (CSF) was collected on three patients, and two had testing for phosphorylated tau (p-tau) and amyloid beta 42 (Abeta42) levels, both of which are valid and sensitive markers for AD pathology. An abnormal CSF ratio of p-tau/Abeta42, in a manner consistent with AD pathology, was noted for both patients. One had slightly elevated CSF protein.

### Treatment

Twenty patients received treatment with medications that included one or more of the following: donepezil, rivastigmine, galantamine, and memantine. Ten patients were treated with donepezil (50%) and nine with memantine (45%). Rivastigmine and galantamine were less commonly used, and six patients were trialed on two or more of the medications noted. Only six respondents answered whether there was a response to medication treatment as determined by *subjective* measures, and two of six reported response as *unknown*, one of six noted the medication was not tolerated (donepezil), one of six noted subjective improvement with donepezil, one of six reported subjective improvement with galantamine and memantine, and one of six noted no benefit with memantine.

### Age Associations

Correlation analyses between age and signs and symptoms revealed statistically significant (*p* < 0.05) negative correlations for limb apraxia (*r* = −0.47) and left–right disorientation (*r* = −0.44). Thus, with older age, patients were less likely at presentation to have limb apraxia and left–right disorientation, which represent functions that, in general, localize to the dominant posterior.

## Discussion

This study of the clinical characteristics of patients with PCA syndrome presenting to the neuro-ophthalmologist was undertaken as a retrospective chart review by 11 neuro-ophthalmologists from 11 institutions and community practice-based clinics. Several important findings deserve highlighting. In particular, the demographics of this cohort show slight differences with prior reports. Our cohort had 74% women, whereas a recent multicenter PCA study by Firth et al. (7) with 117 patients had 61% women. A large, multicenter PCA genetic risk study, by Schott et al. (8), had 59% women in a cohort of 302 patients. Given the 3:1 ratio of women to men in our cohort, it is worthwhile to determine whether women are more likely to be referred to a neuro-ophthalmologist for unexplained visual symptoms due to PCA or if a larger sample size would reveal no differences in gender for PCA presentation to the neuro-ophthalmologist compared to other cohorts. Average age at presentation in our cohort was 67.8 ± 9.70 years with a median of 69 years, and this represents an older age than in published reports, which reveal an average age of <65 years. The study of Schott et al. (8) reported an average age of 58.9 ± 6.9 years, and 60 ± 8.1 years was the average age in the study of Firth et al. (7). The age range of 45–81 years in our cohort is wide, with half of the cohort greater than the median age of 69 years. The reason for an older age at presentation in this cohort is unknown. Interestingly, a study by Millington et al. (13) with 10 patients with PCA recruited from a neuro-ophthalmology clinic in the United Kingdom reported an age range of 53–77 years with a median of 70 years. As with biological sex, age at presentation to the neuro-ophthalmologist deserves further investigation to determine if those patients presenting to a neuro-ophthalmologist are a unique clinical cohort or represent people with PCA with a greater than average delay in diagnosis.

The prevalence of TBI of 21% in this cohort is low given that as many as 40% of the general population report a history of TBI during their lifetime (14). There is controversy as to whether TBI is a definitive risk factor for AD pathology (14), and more work is necessary to understand the relationship between TBI and PCA. Meanwhile, the nature of this study does not allow further conclusions at this stage. With regard to family history, the prevalence of dementia or AD in first-degree relatives of people with PCA is unknown and is infrequently reported in published cohorts. One longitudinal study of 12 patients from Australia noted only one patient (8%) reported a family history of dementia (15), whereas our cohort had more than double that prevalence. Until larger cohort studies collect and report family history, these data are challenging to interpret.

Among presenting symptoms, difficulty reading was the greatest concern by patients on presentation to the neuro-ophthalmologist and was reported by 91%. The published survey of cognitive experts noted that alexia is thought not to be as common at presentation, with most experts rating its frequency as either “rare” (0–25%) or “common” (25–75%) (2). Methodological differences (opinion survey vs. chart review), physician specialty (cognitive specialist vs. neuro-ophthalmologist) influencing history reporting and/or gathering, or a combination of these factors, could have contributed to these differences. Nonetheless, it is possible that patients with difficulty reading are more likely to present to the neuro-ophthalmologist than a cognitive specialist. One study from China that evaluated reading differences between patients with PCA and patients with non-PCA early-onset AD (EOAD) reported that reading issues could differentiate PCA from EOAD, and characteristic issues included missing words (94%), getting lost on the page (86%), and getting lost from one line to the next (67%) (16), which is similar to English language reports, but the problems encountered while reading were not assessed during the clinical consultations of patients in our study.

Memory complaints were commonly reported in our cohort (77%), while the survey of cognitive experts indicates that the frequency of anterograde memory deficit is similar to the frequency of alexia, either “rare” (0–25%) or “common” (25–75%) (2). The differences could be contributed to the older age of our cohort, but there was no statistically significant correlation between age and the presence of a memory complaint in our cohort. Given the differences between methodologies (survey vs. chart review) and the lack of standardized collection of data, these differences might not be significant. Limb apraxia and left–right disorientation were negatively correlated with age in our cohort, and longitudinal studies will be necessary to know how age influences the sign and symptom profile of PCA syndrome at presentation and whether a specific atrophy or dysfunctional network pattern can be recognized as age related. It is interesting to note that many patients with PCA report symptoms of memory dysfunction even when higher-order visual processing deficits are likely to blame for their concerns about memory. For instance, a patient with PCA (not within this cohort) presented with complaints that she had difficulty finding her hamper in her bedroom because she “…could not remember where it was.” Given that this patient had moderate to severe simultanagnosia and higher-order visual dysfunction with only mild memory dysfunction, this complaint was more likely due to an inability to “see” the hamper due to loss of simultaneous perception rather than an inability to recall “where” the hamper was in the bedroom. A recent study of 12 patients with PCA and simultanagnosia revealed that slowed visual processing speed, and not visual short-term memory capacity, explains simultanagnosia (17), and interestingly, atrophy of white matter, specifically the superior longitudinal fasciculus and not gray matter, was linked to slowed processing speed.

In regard to signs on examination, visual field defects (89%), color plate testing abnormalities (88%), and stereovision impairment (86%) were very common. Visual field defects were reported in the previously published expert survey as either “rare” (0–25%) or “common” (25–75%) at presentation (2). This discrepancy is most likely due to methods of clinical assessment, particularly with regard to visual field testing because threshold perimetry is a routine tool employed in the evaluation of patients presenting to the neuro-ophthalmologist, and threshold perimetry is a sensitive measure of diminished stimulus detection as opposed to confrontational visual field testing with finger counting. In support of this is the finding of a similarly high prevalence of visual field defects when threshold perimetry was used in a smaller cohort of PCA patients who presented from behavioral neurology clinics (9). Homonymous defects in our cohort accounted for 62% of visual field defects, and 83% were left-sided. This pattern is consistent with prior studies (10). A recent study of patients with PCA and homonymous visual field defects found that the hemifield defect is associated with contralateral occipital atrophy, as well as contralateral hippocampal and parahippocampal gyrus atrophy and diminished contralateral posterior white matter integrity, specifically the optic radiations contralateral to the field of vision loss (13). Changes in white matter integrity in PCA deserve more attention given these recent findings by Millington et al. (13) and the findings Neitzel et al. (17) regarding the superior longitudinal fascicle and simultanagnosia. It is also noteworthy that only 2 of 38 patients with PCA had unreliable visual field testing. Future investigation regarding the value of threshold visual perimetry as a unique diagnostic biomarker of the PCA syndrome is indicated.

Pseudoisochromatic color vision testing abnormalities were very common and could reflect central achromatopsia, simultanagnosia, or both. Since simultanagnosia was reported in just under 60% of patients, whereas abnormal color plate testing was noted in 88% of patients, then central achromatopsia as a contributing factor is possible, particularly because the presence of simultanagnosia was not significantly correlated to color plate testing abnormalities in this cohort. However, 13 of 25 patients tested with pseudoisochromatic color plates were unable to see the control plate, which should be identifiable to those with achromatopsia but without simultanagnosia. Further investigation using color vision tests that do not rely on identification of an embedded figure will be necessary to better understand the frequency of central achromatopsia at presentation with PCA and the utility of central achromatopsia as a clinical marker of PCA. Impaired stereopsis was also common in this PCA cohort, and this could be due to a variety of different causes, but data regarding monocular visual acuities and history of amblyopia were not collected. The high prevalence of impairment, nonetheless, deserves further investigation in order to determine whether the underlying cause for this sign stems from poor central fusional capacity.

Central vision crowding was detected in more than half of the 20 patients whose crowding status at presentation was known, and visual crowding is a well-recognized phenomenon of the PCA syndrome. In the neuro-ophthalmology clinic, evaluation for crowding is typically assessed by comparing visual acuity using letters flanked by other letters to visual acuity using letters that are non-flanked (or isolated). As noted, however, a history of amblyopia was not surveyed, and a recent population-based study found a prevalence of amblyopia is ~7% in people between 55 and 65 years old (18). Recent work by de Best et al. (19) sheds light on the potential mechanism of central vision crowding in PCA. Investigators found that population receptive fields (pRF) in V1 (striate cortex) and V4 (including lingual and fusiform regions) are larger in foveal regions and smaller in peripheral regions than in healthy controls. The authors theorized that because neurons dedicated to foveal vision suffer from an impaired ability to properly process a stimulus that is crowded by other stimuli when their pRF enlarges, central vision crowding follows. With shrinking of the pRF for peripheral (non-foveal) neurons, local stimulus features are processed at the expense of global features, and the result is simultanagnosia. These findings and theory deserve further exploration and have implications for understanding crowding in other disorders. Reduced visual acuity was more common (60%) in our cohort than the survey by experts, and again, this is likely due to the methodology (survey of impressions vs. chart review), subspecialty assessment (cognitive specialists vs. neuro-ophthalmologists), or older age of patients. Until further investigations occur, the reasons will remain unknown.

Brain imaging was most commonly reported as “generalized atrophy,” and it behooves the clinician to review the imaging for signs of posterior greater than anterior atrophy and consider PET-FDG imaging, as findings indicated posterior predominant atrophy in nearly all cases. In regard to treatment, although medications are commonly prescribed, there are no known effective pharmaceutical therapies for the PCA syndrome.

The major limitation of this study is that it is a retrospective chart review of clinical data that were not collected using a standardized assessment protocol.

## Conclusion

The presentation of PCA to the neuro-ophthalmologist in this cohort is slightly different than the presentation reported in published cohorts and reported by cognitive specialists. Symptoms and signs at presentation are comparable, but patients presenting to the neuro-ophthalmologist with PCA are more likely to be older than 65 years, female, and with concerns about reading. Furthermore, visual field defects, pseudoisochromatic color vision test abnormalities, and abnormal stereopsis were very common, and visual field tests were performed reliably by nearly all patients. Consistent with other investigations of PCA, left-sided, homonymous visual field defects were the most common field abnormality. The frequency of these abnormal signs likely reflects the unique evaluation performed within a neuro-ophthalmology clinic and reveals potential to be useful markers of the PCA syndrome.

Neuro-ophthalmologists play a critical role in diagnosing PCA and can contribute to a better understanding of the syndrome. Future investigations of PCA should focus on longitudinal, standardized data collection from multiple institutions and centers, with involvement of a wide spectrum of subspecialists, including those practicing in the community. Early recognition and a better understanding of progression of the PCA syndrome will benefit patients, families, and care partners and contribute to our understanding of disease mechanisms and development of new therapies.

## Resource Identification Initiative

REDCap, RRID:SCR_003445.

## Data Availability Statement

The raw data supporting the conclusions of this article will be made available by the authors, without undue reservation, to any qualified researcher.

## Ethics Statement

The studies involving human participants were reviewed and approved by the OHSU Institutional Review Board. Written informed consent for participation was not required for this study in accordance with the national legislation and the institutional requirements.

## Author Contributions

JO and WH contributed to the conception or design of the work and oversaw the statistical analysis. VP, JO, and WH analyzed and interpreted the data, and wrote and revised the manuscript. JW, JF, LA, MM, RE, WC, AL, BF, RT, DK, and JC contributed to the acquisition of data for the work. All authors contributed to manuscript read and approved the submitted version.

## Conflict of Interest

The views expressed are those of the author and do not reflect the official policy or position of the US Air Force, Department of Defense, Defense Health Agency, or the US Government. The authors declare that the research was conducted in the absence of any commercial or financial relationships that could be construed as a potential conflict of interest.
